# Oxygen Loss on Disordered Li-Excess, Mn-Rich Li-Ion Cathode Li_2_MnO_2_F through First-Principles Modeling

**DOI:** 10.1021/acs.chemmater.3c01815

**Published:** 2023-10-23

**Authors:** Jordan Burns, Kristin A. Persson

**Affiliations:** † Energy Storage and Distributed Resources Division, Lawrence Berkeley National Laboratory, Berkeley, California 94720, United States; ‡ Department of Materials Science and Engineering, University of California, Berkeley, California 94720, United States; § Molecular Foundry, Energy Sciences Area, Lawrence Berkeley National Laboratory, Berkeley, California 94720, United States

## Abstract

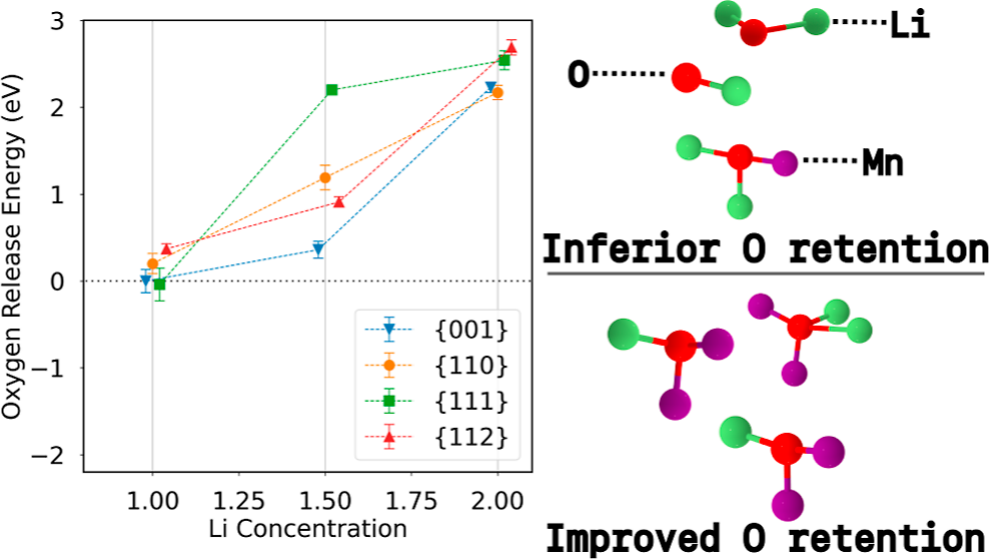

Oxygen evolution energies were calculated for the {100}, {110}, {111}, and {112} type facets of the rock-salt-structured cation-disordered Li-excess, Mn-rich Li-ion (DRX) cathode Li_2_MnO_2_F at the fully lithiated, 25% delithiation, and 50% delithiation states. Our calculations showed that Li_2_MnO_2_F remains much more robust to O loss than its nonfluorinated counterparts, as has been shown in experimental work. In particular, the {110} and {112} facets are the most resilient against O loss. Focusing on the {100} type facet, which previous work has shown to be the most likely exposed facet of Li_2_MnO_2_F, it was found that higher proportions of Li in an O coordination shell lead to lower O evolution energy (*Ẽ*_O_) and facilitate O loss. It was also found that at higher states of delithiation, surface fluorine had a weaker effect in increasing *Ẽ*_O_, meaning the protective effect of F against O loss is more effective at higher lithiation states. Electronic structure chemical bonding analysis revealed weaker bonding interactions between Li and O correlated with lower *Ẽ*_O_ and hence a higher propensity for surface O loss..

## Introduction

Given the urgent need to shift consumption from nonrenewable fossil-fuels toward sustainable energy resources,^1^ the demand for energy storage is rapidly increasing and projected to continue increasing.^2^ Disordered rock-salt Li-excess (DRX) cathode materials^3−5^ have emerged as a promising candidate for Li-ion battery applications, offering a wide chemical space allowing for the use of cheap, earth-abundant materials. Fluorination has been shown to improve the performance of DRX materials,^6−10^ simultaneously increasing the transition metal redox capacity and mitigating the capacity and voltage fade associated with surface O loss.

Both DRX materials^11−13^ as well as cation-ordered, layered materials^14,15^ have been observed to exhibit O loss. To investigate the chemical nature of the bulk O loss, Seo et al.^16^ examined Li-rich layered oxides and DRX cathodes with density functional theory (DFT) and highlighted that the O 2p orbital in Li–O–Li configurations remains unhybridized. The lack of hybridization in turn leads to an increased O energy state and liability toward O oxidation at high charge. Other studies have shown the formation of peroxo-like species^17^ as well as transition metal migration^18^ correlated with voltage fade. Shin and Persson^19^ predicted the thermodynamic driving force for O evolution to increase with delithiation in the layered material Li_2_MnO_3_. In particular, they noted that while the release of O already becomes spontaneous at 25 and 50% states of delithiation for all facets studied, the (110) surface is most resistant to O loss. Hu et al. studied various cathode materials including layered LiMO_2_ (M = Co, Ni, Mn) and LiNi_1/3_Co_1/3_Mn_1/3_O_2_ (NCM-333), spinel LiMn_2_O_4_, and Li-rich Li_2_MnO_3_ and found that all materials except for Li_2_MnO_4_ were more likely to lose O after delithiation.^20^ Using computational methods and focusing on the DRX material Li_2_MnO_2_F, Sharpe et al.^21^ showed that 5 Li-, 1 Mn-coordinated O were more prone to oxidation upon charge as compared to those surrounded by only 3 or 4 Li. They also found, using a combination of computations and RIXS spectroscopy, that at high levels of Li deintercalation, molecular O_2_ is energetically favored to form within the bulk of the material, and O_2_ to be reversibly reduced to O^2–^ on discharge. Surface reconstruction and phase changes have also been observed following O loss. Using first-principles based Monte Carlo simulations, Xiao et al.^22^ suggested that densified phases of the types Ni_0.25_NiO_2_ and Ni_0.5_NiO_2_ can form by Ni back diffusion from the surface owing to O loss at highly charged states. Interestingly, they also found that slow kinetics preclude the conventional Li_0.5_NiO_2_ spinel formation from its layered parent phase at room temperature. Mukherjee et al.^23^ leveraged electron microscopy and spectroscopy to study the effects on O loss in LiNi_0.8_Co_0.15_Al_0.05_O_2_ (NCA) cathodes held at 4.75 V and 60 °C. They observed phase transformations from layered to rock salt followed by metal dissolution leading to the irreversible formation of a porous surface layer with high impedance. Kwon et al.^24^ found that following O loss, both Li_1.2_Ni_0.333_Ti_0.333_Mo_0.133_O_2_ (LNTMO) and Li_1.2_Mn_0.6_Nb_0.2_O_2_ (LMNO) experience metal densification at the surface. However, LMNO forms a surface phase that allows fast Li transport and alleviates further loss of O, whereas LNTMO’s densified DRX surface structure does neither. In the DRX material Li_2_MnO_2_F, McColl et al.^25^ found that irreversible transition metal migration is required before molecular O_2_ can form in the bulk structure, such that O^2–^ ions move to sites with different coordination causing a voltage fade corresponding to 0.3 V.

Responding to the detrimental effect of O loss, mitigation strategies have been proposed. Craften et al.^10^ suggested that fluorination can completely suppress O loss in the DRX material Li_1.2_Mn_0.625_Nb_0.175_O_1.95_F_0.05_ when cycled between 1.5 and 4.45 V. Even increasing the charging protocol to 4.8 V showed only a small amount of O evolution on the first cycle and none thereafter. Park et al.^26^ employed a graphene-based hermetic surface coating that reduced O loss in layered LiNiO_2_ at high states of charge. Shin et al.^27^ used DFT to study the effect of surface dopants on the oxygen evolution energy (*Ẽ*_O_) in layered Li_2_MnO_3_ and found a beneficial effect from Os, Sb, Ru, Ir, and Ta, while Ti and Al did not improve O retention. The authors explained the improved O retention through an increase in the hybridization of p-orbitals between the O and dopant atoms. Experimental differential electrochemical mass spectroscopy confirmed that Ta-doping (Li_1.3_Nb_0.285_Ta_0.015_Mn_0.4_O_2_) reduced O evolution compared to undoped Li_1.3_Nb_0.3_Mn_0.4_O_2_. Ning et al.^28^ found that a defective graphene coating can suppress O loss on Li_2_MnO_3_ by modifying the potential energy surface.

Further computational work studying the energetics of O loss of oxygen on DRX surfaces could lead to design rules to help synthesize DRX cathodes with increased resistance to O loss. Here, we use the highly fluorinated Li-excess, Mn-rich DRX cathode Li_2_MnO_2_F as a model system to study the thermodynamic driving force for O release. Previously, the low Miller index surfaces of Li_2_MnO_2_F were studied using computational methods, which revealed the {100} type surface as the lowest-energy facet, which hence dominated the equilibrium particle shape. It was also found that the surface was enriched in Li and F.^29^ Following these results, we here calculate the O release energy (*Ẽ*_O_) on the low Miller index surfaces {100}, {110}, {111}, and {112} at 0, 25, and 50% levels of delithiation. We base our disordered structures on a cluster expansion method described in Ouyang et al.^30^ as well as a surface-symmetrizing procedure described in Burns et al.^29^ For each facet and delithiation level, we calculate the *Ẽ*_O_ on surfaces of varying chemistry and arrangements (guided by the aforementioned findings on the preferred surface chemistry) and use Boltzmann weighting to get average O release energies.

## Methods

### DFT Calculations

All first-principles calculations were performed using the Vienna ab initio simulation package (VASP)^31−34^ using the projector augmented-wave potentials^35^ with the Perdew–Burke–Ernzerhof generalized-gradient approximation functional.^36^ Structure optimizations were used to fully relax the atomic positions and lattice parameters in the bulk structures. Slab optimizations were used to fully relax the atomic positions of the 3 outermost surface layers of slabs using at least 20 Å of vacuum in both pristine and O deficient slabs. All bulk and slab total energies were then improved by using static calculations. The max force cutoff was relaxed from 0.25 to 1 eV/Å for the 50% delithiated structures for all facets, as well as the 25% delithiated {100}, {110}, and {111} structures. Static calculations were performed prior to the chemical bonding analysis using the software package LOBSTER.^37−40^ All INCAR parameters used were bench-marked by the Materials Project and are documented in MPRelaxSet, MPStaticSet, and LobsterSet in Python Materials Genomics (pymatgen).^41^

### Oxygen Evolution Energy Workflow

All bulk structures were generated using Monte Carlo simulations based on a previously trained cluster expansion.^30^ Structures were equilibrated with at least 100,000 Monte Carlo steps and then sampled during a production run of at least 100,000 Monte Carlo steps at a temperature of 2573 °C. Symmetric surfaces were generated from the sampled structures as described in Burns et al.^29^ Bulk structure relaxations were performed, and the resulting relaxed structures were used to create supercells of dimensions 1 × 1 × 2. The relaxed bulk structures were used in the pristine slab calculations. To create O deficient slabs, one O atom was removed from the outermost top and bottom layer of relaxed pristine slabs, and a slab calculation was performed on the resulting O deficient slab. Slab dimensions in both the *x*- and *y*-directions were created at a minimum of 8 Å in order to avoid defect–defect interactions by the removed O and its periodic images.

### Delithiating Structures

To identify low-energy Li vacancy orderings, we trained an L2 regularized least-squares regression model on total energies obtained from 33 DFT calculations of slabs with random orderings of Li vacancies, achieving a root mean squared error of 11 meV per atom. The regression model predicts an increase in the energy for clusters of Li vacancies and vacancy-F coordination. Furthermore, it predicts a lowering in energy for undercoordinated surface Li vacancies, in agreement with prior work.^42^ To sample structures with reasonable vacancy decorations, at least 1000 random Li vacancy orderings were generated, and our model was used to predict the energy of these structures. The final selected structures were sampled from the lowest predicted energy orderings.

### Surface and Oxygen Evolution Energy Calculations

Surface energies were calculated as follows
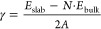
1where γ is surface energy, *E*_slab_ is the total energy output of the pristine slab calculation, *N* is the number of bulk layers in the supercell (two for all calculations here), *E*_bulk_ is the total energy of the bulk calculation of the parent bulk structure of each specific pristine slab, and *A* is the surface area of the pristine slab.

The *Ẽ*_O_ was calculated according to the following equation
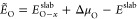
2where  is the total energy of the O deficient slab, Δμ_O_ is the corrected O chemical potential as calculated by Wang et al. and Aykol et al.^43,44^ and *E*^slab^ is the total energy of the pristine slab.

### Boltzmann Weighted Oxygen Evolution Energy Calculations

To obtain a single value of the *Ẽ*_O_ for each facet and delithiation state, Boltzmann weighting is performed according to the following equation
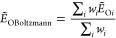
3where *Ẽ*_OBoltzmann_ is the Boltzmann weighted *Ẽ*_O_ for a given facet and lithiation state, *Ẽ*_O*i*_ is the *Ẽ*_O_ of calculation *i* and *w*_*i*_ is defined as
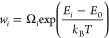
4where Ω_*i*_ is the multiplicity of surface *i*, *E*_*i*_ is the energy per atom of the pristine slab, *E*_0_ is the lowest per atom slab energy calculated for the given facet and delithiation state, *k*_B_ is the Boltzmann constant and *T* is the temperature. The multiplicities were calculated similarly to the method in Burns et al.^29^ Statistics for each facet were taken from at least 1,000,000 structures generated by Monte Carlo. Laplace smoothing with an α value of 1 was applied to all multiplicities. As the number of possible surfaces is intractably large for direct calculation by DFT, we use a sample of the population of possible surfaces to estimate the *Ẽ*_O_ on each facet and bootstrap our results to explore the possible ranges of energies that might have been found with a different sample.

### Bonding Analysis

In order to analyze the bonding and antibonding character between the removed O atom and its nearest neighbor atoms, a Crystal Orbital Hamiltonian Population analysis was performed using the Local-Orbital Basis Suite Toward Electronic-Structure Reconstruction (LOBSTER) package.^37−40^ LOBSTER input parameters were generated using pymatgen, and all interactions within 6 Å were calculated. Results were obtained with less than 1% charge spilling, indicating that LOBSTER was able to transfer over 99% of the charge density from the occupied levels of the original wave functions obtained from DFT into the local basis chosen.

## Results and Discussion

**Figure 1:**
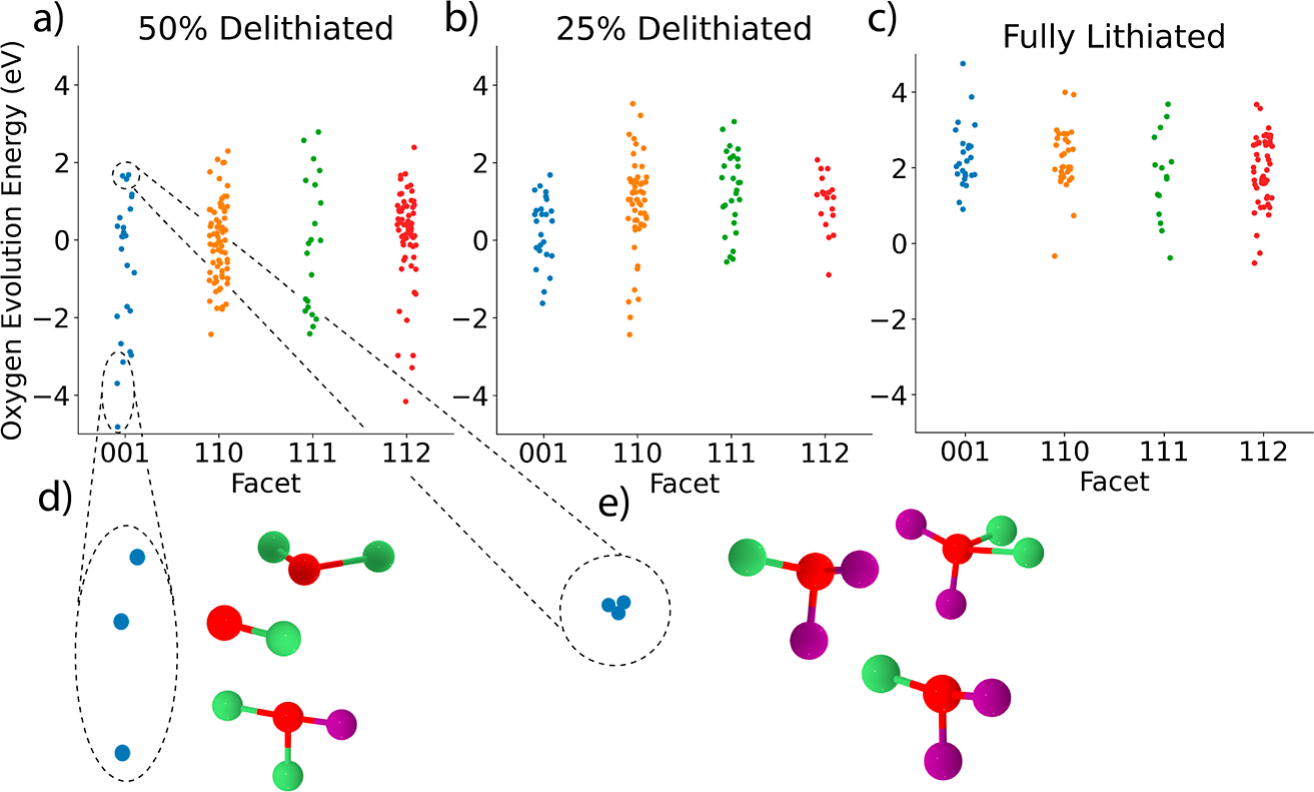
Calculated *Ẽ*_O_ are shown for the {100}, {110}, {111}, and {112} type facets of DRX Li_2_MnO_2_F for the (a) 50% delithiated state Li_1.0_MnO_2_F, (b) 25% delithiated state Li_1.5_MnO_2_F, and (c) the fully lithated state Li_2_MnO_2_F. A small amount of jitter was added in the *x* direction for each point to ease viewing of the data. (d) The three lowest *Ẽ*_O_ and their corresponding coordination environments and (e) the three highest *Ẽ*_O_ and their coordination environments for the {100} type surface at the 50% delithiation state. Red atoms represent O, green atoms represent Li, and purple atoms represent Mn.

*Ẽ*_O_ were calculated for the {100}, {110}, {111}, and {112} type facets of DRX Li_2_MnO_2_F at three lithiation states: 0, 25, and 50% delithiation. In total, 439 *Ẽ*_O_ were calculated through first-principles methods. Figure 1 shows the energy spread of calculated *Ẽ*_O_ for each facet and lithation state. Across facets, there is a consistent trend in decreasing *Ẽ*_O_ as a function of decreasing Li content within Li_2_MnO_2_F.

Previous work has shown that the equilibrium particle shape of Li_2_MnO_2_F is dominated by {100} type facets.^29^ O evolution has also been shown to more severely affect cathode materials at deeper states of delithiation.^10^ Hence, we choose the {100} type facet at the deepest delithiation level of 50% for a closer study. Selecting the three O ions with the highest/lowest *Ẽ*_O_, we examine the coordination environment, which is shown as an inset (d,e) in Figure 1. We find that the O with the highest evolution energies, *Ẽ*_O_, are relatively highly coordinated with a larger representation of Mn as nearest neighbors. In contrast, the O with the lowest *Ẽ*_O_ are relatively sparsely coordinated and are mostly or entirely coordinated with Li.

**Figure 2:**
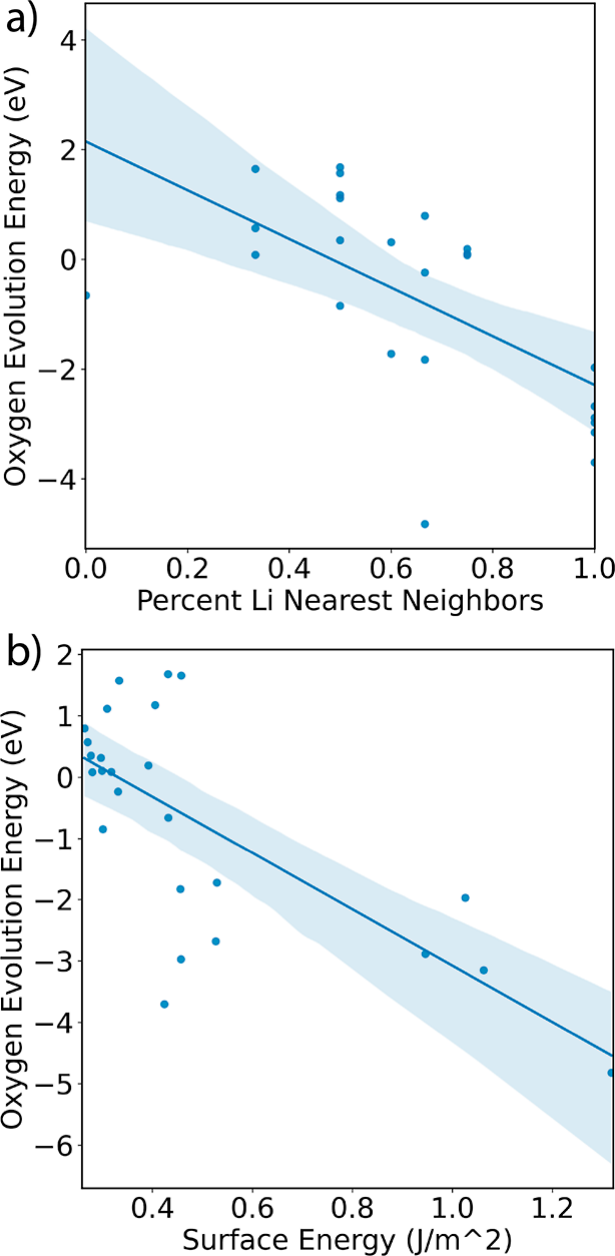
Lines of best fit are shown for (a) the relationship between *Ẽ*_O_, and the percentage of Li nearest neighbors for the considered O and (b) the energy of the surface from which the O was removed. The light blue regions show a 95% confidence interval for the lines of best fit by bootstrapping the data resampling 1000 times. These data are both from the {100} type surface at the 50% delithiation state of Li_1.0_MnO_2_F.

Inspection of the *Ẽ*_O_, calculated for the {100} type surface at the 50% delithiation state, Figure 2a shows that there is a strong relationship between the *Ẽ*_O_ and the proportion of Li-ions in the O coordination shell. A line of best fit and 95% confidence interval were calculated using Seaborn^45^ and Scikit-learn^46^ which predict that a change from 0 to 100% coordination by Li corresponds to a significant *Ẽ*_O_ decrease of −4.43 eV. Similarly, and aligned with the findings of Seo et al.^16^ that Li–O–Li exhibit unhybridized O_p_ states, the lowest *Ẽ*_O_ in Figure 1d,e correspond to O almost entirely coordinated by Li and the highest *Ẽ*_O_ correlate with relatively high occurrence of nearest neighbor Mn. Figure 2b shows that as the surface energy of a given surface increases, the *Ẽ*_O_ decreases. Using Seaborn and Scikit-learn to create a line of best fit, the data predicts that for the {100} type surface at the 50% delithiation state, an increase in surface energy of 1 J/m^2^ would cause a corresponding, significant decrease of −4.60 eV. The surface energy provides a general measure of the stability of the surface, and hence, we expect that higher surface energies should correlate with a higher tendency toward O loss.

**Figure 3:**
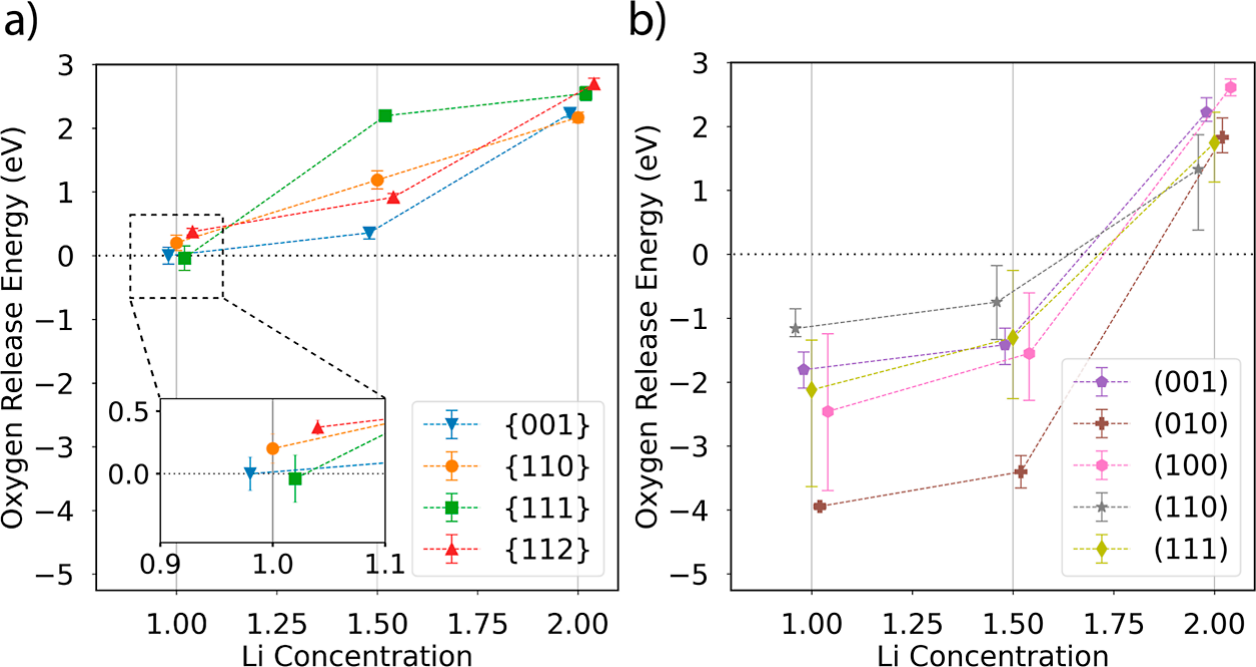
(a) Boltzmann weighted *Ẽ*_O_ are shown for all facets and lithiation states. (b) A corresponding data set for the ordered, layered material Li_2_MnO_3_ showing the average *Ẽ*_O_ is shown for comparison (adapted from Shin, Y.; Persson, K. A. Surface morphology and surface stability against oxygen loss of the lithium-excess Li_2_MnO_3_ cathode material as a function of lithium concentration. *ACS Appl. Mater. Interfaces 2016,***8,** 25595–25602. Copyright [2016] American Chemical Society). A small amount of jitter was added to the *x*-value of each data point to ease viewing of the data, but each point was calculated at exactly the fully lithiated, 25 or 50% delithiation state. Error bars in (a) were calculated by bootstrapping the data resampling 1000 times and taking the standard deviation of the resulting samples. A magnification of the *Ẽ*_O_ at the 50% delithiation state from the area indicated by the dashed box is shown in an inset in (a).

Figure 3 depicts the Boltzmann weighted *Ẽ*_O_ for Li_2_MnO_2_F for each facet at all three levels of lithiation next to the average *Ẽ*_O_ found by Shin and Persson^19^ for the layered cathode material Li_2_MnO_3_. An area of inset (a) is magnified to more clearly show the cluster of points at the 50% delithiation state. Table 1 shows the values for each Boltzmann weighted *Ẽ*_O_ of Li_2_MnO_2_F. While the *Ẽ*_O_ quickly fall below zero in the case of Li_2_MnO_3_ as delithiation proceeds, the average *Ẽ*_O_ remain positive for DRX Li_2_MnO_2_F, even at the 50% delithiation state, where just the {100}- and {111}-type facets approximately attain zero. Indeed, prior work^10^ supports our findings here by showing a strong correlation between F substitution for O and increased O retention.

**Table 1:** Boltzmann Weighted *Ẽ*_O_ for all Facets and Lithiation States at 298 K

Boltzmann weighted *Ẽ*_O_(eV)			
facet	50%	75%	100%
{100}	–0.001	0.36	2.23
{110}	0.20	1.19	2.17
{111}	–0.04	2.20	2.54
{112}	0.37	0.91	2.69

**Figure 4:**
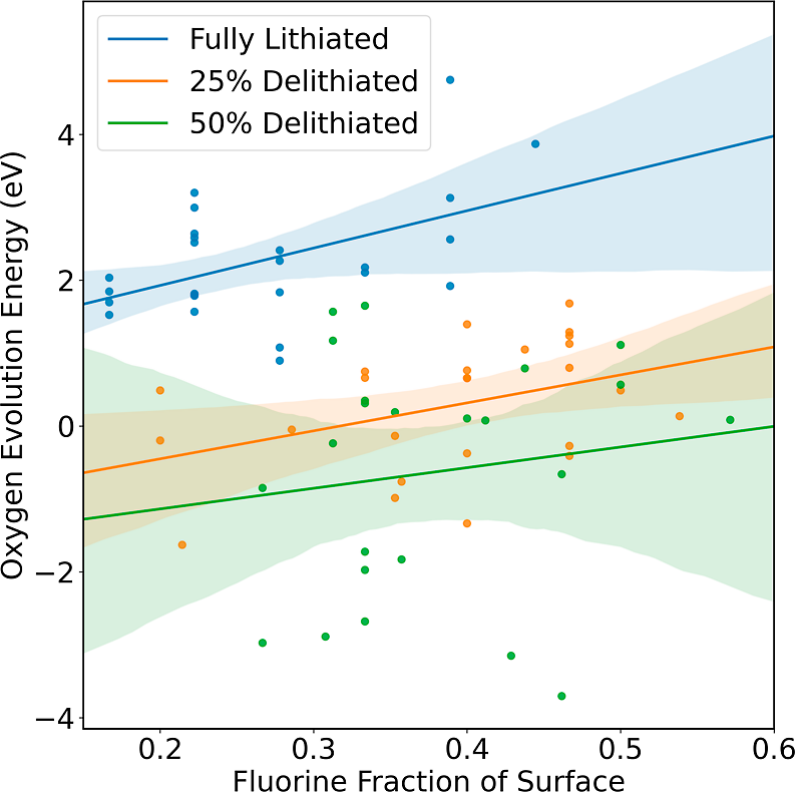
Lines of best fit are shown for the relationship between *Ẽ*_O_, and the percentage of surface fluorine for the {100} surface at the fully lithiated, 25% delithiated and 50% delithiated states. The light colored regions surrounding the lines of best fit show a 95% confidence interval for the lines of best fit by bootstrapping the data resampling 1000 times.

Substituting O with F atoms has been shown to reduce O loss at the surface of DRX materials through multiple mechanisms. As reported in previous studies,^47,48^ substituting O^2–^ ions with F^–^ ions lowers the valence state of the Mn ions, allowing for more Mn redox and minimizing the participation of O^2–^ for the same delithiation state. Second, Li–F surface decoration is more stable than O–Mn in Li_2_MnO_2_F, which means that surface oxygen is simply less abundant^29^ and, as shown here, reduces the oxygen release energy, *Ẽ*_O_. Here, we also investigate the role of F on the surface of Li_2_MnO_2_F in reducing the oxygen loss across different states of delithiation. Figure 4 shows the relationship between the percent of surface fluorine and *Ẽ*_O_. This relationship is investigated for the {100} surface at the fully lithiated, 25% delithiated, and 50% delithiated states. While there is clearly a large difference in protection against O loss by the presence of F when comparing fluorinated Li_2_MnO_2_F with its unfluorinated, layered counterpart Li_2_MnO_3_ as shown in Figure 3, Figure 4 shows that there is some variation in this protection from O loss which depends on the lithiation level. Lines of best fit and 95% confidence intervals were calculated using Seaborn^45^ showing that for all three delithiation levels, increasing levels of surface F predict a higher *Ẽ*_O_. However, calculating the slopes of these lines of best fit using Scikit-learn,^46^ we found the fluorine stabilizing effect subsides at higher levels of delithiation. A change from 0% surface *F* to 50% surface *F* at the fully lithiated state predicts an increase in *Ẽ*_O_ of 2.6 eV, whereas the predicted increase in *Ẽ*_O_ is only 1.9 eV for the 25% delithiation state and 1.1 eV for the 50% delithiation state. As delithiation proceeds, an increasing fraction of surface F is required to confer protection against oxygen loss.

**Figure 5:**
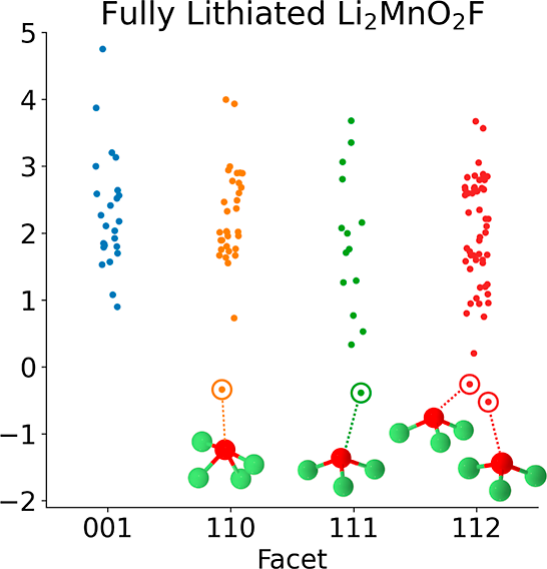
Coordination environments are shown for the O with evolution energies below 0 eV at the fully lithiated state Li_2_MnO_2_F. Red atoms represent the O, and green atoms represent Li.

There are a few surfaces with negative *Ẽ*_O_ even in their fully lithiated state. Examining these cases (see Figure 5), we find that the surface O pertaining to these specific data points is entirely coordinated by Li. Previous experimental work^10^ showed that fluorinated DRX cathodes do lose a small amount of O during their first cycle and then passivate against further O loss. We hypothesize, from our findings and in agreement with prior work that shows low O hybridization for Li–O–Li environments, that the O that are lost upon the first cycle are highly coordinated by Li.

**Figure 6:**
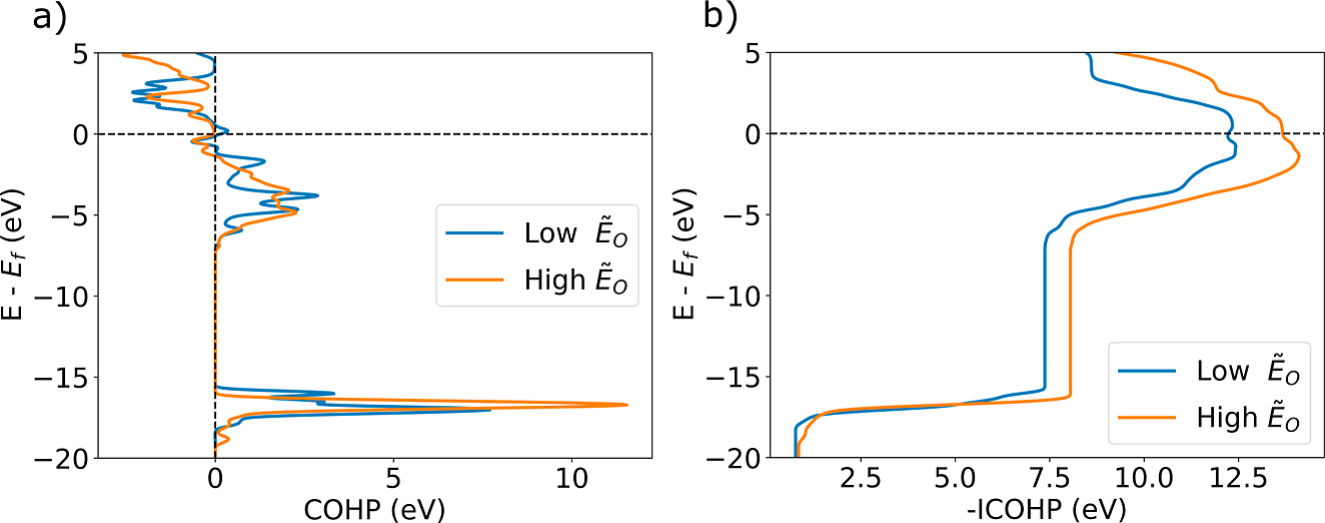
Total (a) COHP and (b) –ICOHP are shown for the highest and lowest *Ẽ*_O_ calculated on the {100} type surface at the 50% delithiation state.

LOBSTER was performed to analyze the bonding interactions of the O atom with its nearest neighbors for the O atoms with the highest and lowest *Ẽ*_O_ calculated on the {100} type surface at the 50% delithiation state. Figure 6 shows (a) the total crystal orbital Hamiltonian population (COHP) and (b) total negative integrated crystal orbital Hamiltonian population (–ICOHP) for the highest and lowest *Ẽ*_O_ calculated on the {100} type surface at the 50% delithiation state. The total includes all of the bonding interactions between the removed O atom and all of its nearest neighbors. The total COHP in (a) shows that the highest *Ẽ*_O_ has more positive bonding interactions in both the deeper core states around −15 to −20 eV as well as some states just below the Fermi energy around −3 to −4 eV. As would be expected in b), the value of the –ICOHP has a larger value at the Fermi energy for the highest *Ẽ*_O_, indicating stronger bonding between the removed O atom and its nearest neighbor atoms.

**Figure 7:**
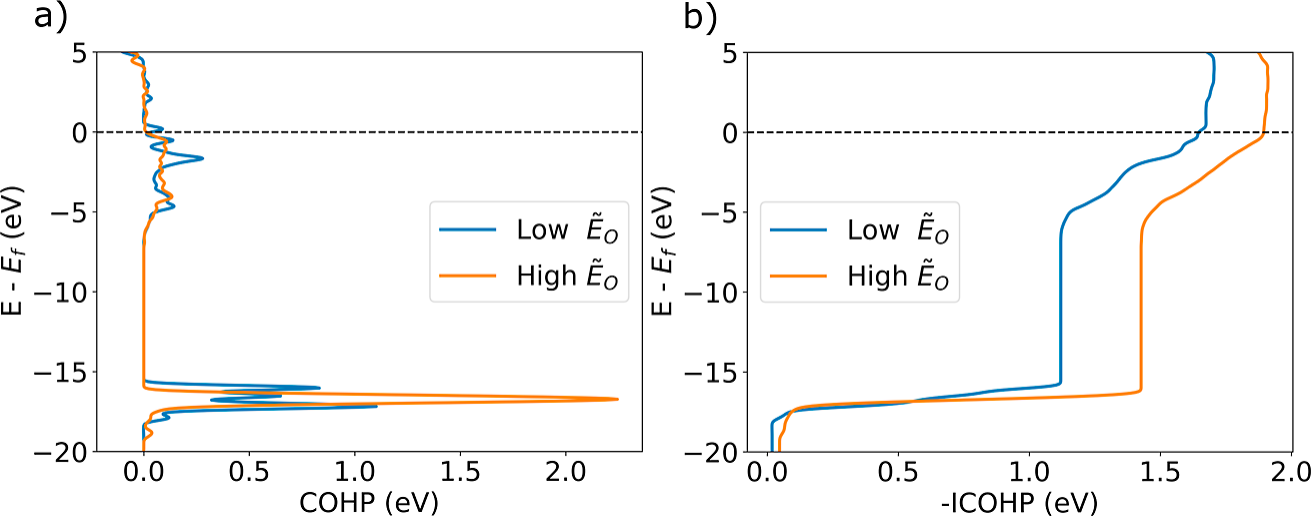
Per-Li atom (a) COHP and (b) –ICOHP between the removed O and its nearest Li neighbors are shown for the highest and lowest *Ẽ*_O_ calculated on the {100} type surface at the 50% delithiation state.

Prior work on bulk DRX and Li-excess layered cathodes have shown low O hybridization for Li–O–Li environments, which motivates a comparison with surface Li–O bonding interactions and associated trends for the *Ẽ*_O_. Figure 7 shows (a) the per-Li COHP and (b) the per-Li –ICOHP for the removed O atom and its nearest Li neighbors. Similar to the total COHP, the per-Li COHP shows that the O with the highest *Ẽ*_O_ exhibit more positive bonding interactions with its nearest neighbor Li atoms in the deeper core state region than the lowest *Ẽ*_O_ removed O. From Figure 7b, we observe that the per-Li atom –ICOHP has a lower value for the lowest *Ẽ*_O_ at the Fermi level, indicating weaker bonding between the removed O atom and its nearest neighbor Li atoms. This weaker Li–O bond correlating with a lower *Ẽ*_O_, and hence a propensity to lose surface O, agrees with previous findings showing that weak Li–O interaction at higher levels of charge leads to O loss at the surface of DRX materials.

From Figure 3a, we observe that, while the Boltzmann weighted *Ẽ*_O_ are very similar for all facets studied at the 50% delithiation state (*x*_Li_ = 1.00), the {112} and {110} remain the most robust to O loss. We note that previous work^29^ showed that the thermodynamic equilibrium particle shape for DRX Li_2_MnO_2_F is dominated by {100} type surfaces and enriched in Li/F. In light of the findings here that Li-coordinated, surface-exposed O on {100} is less resilient against gassing during delithiation, there may be interest in exploring different synthesis conditions and their respective resulting particle morphologies; particularly with the goal of maximizing the exposure of {112} and {110} surface facets.

## Conclusions

Over 400 first-principles calculations and Boltzmann weighted oxygen evolution energies (*Ẽ*_O_) for 159 unique surface terminations of the four majority facets; {100}, {110}, {111}, and {112} type surfaces at the fully lithiated, 25% delithiated, and 50% delithiated states of DRX Li_2_MnO_2_F are presented. In agreement with previous work, *Ẽ*_O_ drop as delithiation progresses. However, in contrast to chemically similar layered and nonfluorinated materials, DRX Li_2_MnO_2_F is predicted to remain much more robust to O loss. At the 50% delithiated state, the {110} and {112} facets were the most resilient to O loss. Examining the {100} surface, it was found that a higher degree of Li coordination correlates strongly with thermodynamic susceptibility toward O loss. The role of surface fluorine in protecting against O loss was also examined. While fluorine generally stabilizes against oxygen loss, we found that the effect subsides as a function of delithiation, for example higher levels of surface of fluorine is needed to achieve the same effect on *Ẽ*_O_ at higher states of charge. The cases with highest and lowest *Ẽ*_O_ were examined for electronic structure trends, and their bonding analyzed with the LOBSTER software. Higher *Ẽ*_O_ was shown to have an overall higher –ICOHP at the Fermi level which indicates overall stronger bonding. In agreement with previous work examining Li–O bonding, our LOBSTER results show that a lower per-Li –ICOHP at the Fermi level between the removed O and its Li nearest neighbors, and hence weaker bonding interactions, is correlated with a lower *Ẽ*_O_ and therefore increases the risk of surface O loss.
